# Disease Dynamics and Bird Migration—Linking Mallards *Anas platyrhynchos* and Subtype Diversity of the Influenza A Virus in Time and Space

**DOI:** 10.1371/journal.pone.0035679

**Published:** 2012-04-20

**Authors:** Gunnar Gunnarsson, Neus Latorre-Margalef, Keith A. Hobson, Steven L. Van Wilgenburg, Johan Elmberg, Björn Olsen, Ron A. M. Fouchier, Jonas Waldenström

**Affiliations:** 1 Division of Natural Sciences, Kristianstad University, Kristianstad, Sweden; 2 Section for Zoonotic Ecology and Epidemiology, School of Natural Sciences, Linnaeus University, Kalmar, Sweden; 3 Wildlife and Landscape Science, Environment Canada, Saskatoon, Canada; 4 Section of Infectious Diseases, Department of Medical Sciences, Uppsala University Hospital, Uppsala, Sweden; 5 Department of Virology, Erasmus Medical Center, Rotterdam, The Netherlands; Centers for Disease Control and Prevention, United States of America

## Abstract

The mallard *Anas platyrhynchos* is a reservoir species for influenza A virus in the northern hemisphere, with particularly high prevalence rates prior to as well as during its prolonged autumn migration. It has been proposed that the virus is brought from the breeding grounds and transmitted to conspecifics during subsequent staging during migration, and so a better understanding of the natal origin of staging ducks is vital to deciphering the dynamics of viral movement pathways. Ottenby is an important stopover site in southeast Sweden almost halfway downstream in the major Northwest European flyway, and is used by millions of waterfowl each year. Here, mallards were captured and sampled for influenza A virus infection, and positive samples were subtyped in order to study possible links to the natal area, which were determined by a novel approach combining banding recovery data and isotopic measurements (δ^2^H) of feathers grown on breeding grounds. Geographic assignments showed that the core natal areas of studied mallards were in Estonia, southern and central Finland, and northwestern Russia. This study demonstrates a clear temporal succession of latitudes of natal origin during the course of autumn migration. We also demonstrate a corresponding and concomitant shift in virus subtypes. Acknowledging that these two different patterns were based in part upon different data, a likely interpretation worth further testing is that the early arriving birds with more proximate origins have different influenza A subtypes than the more distantly originating late autumn birds. If true, this knowledge would allow novel insight into the origins and transmission of the influenza A virus among migratory hosts previously unavailable through conventional approaches.

## Introduction

Migration is a common feature of birds living in seasonal environments, and movements range from very short altitudinal movements to trans-hemispherical flights of tens of thousands of kilometres [1], [2]. Bird migration potentially facilitates the spread of pathogens at multiple geographic scales, and could consequently influence disease dynamics [3]–[5]. This is particularly evident for populations of the same species separated during one part of the year but co-occurring in another (cf. “migratory connectivity” in [6]), which not only facilitates population mixing and the maintenance of genetic diversity [6]–[8], but is also likely of great importance for disease transmission [5]. Temporal and spatial variation in the dynamics of disease in relation to migration is a rather young area of research. At present, there are a few model systems in this field, of which influenza A virus dynamics in waterfowl is one. Influenza A viruses are negative-sense RNA viruses capable of infecting a broad range of hosts, most commonly avian, but also mammalian hosts, including man [9]. Dabbling ducks (*Anas* spp.), and in particular the mallard *A. platyrhynchos*, have been implicated as the most important reservoir and vector for low-pathogenic avian influenza (LPAI) viruses [9]. Most of the described virus subtypes have been recorded in wild mallards and virus prevalence appears to vary predictably by season and geography [9]–[12]. The virus infects epithelial cells of the gastrointestinal tract and the bursa, but does not appear to induce pathogenic lesions in those tissues [13]. However, studies indicate that influenza A virus infection in mallards could translate into an ecological cost, manifested in reduced body mass [14]. More considerable effects have been observed in wintering Bewick's swans *Cygnus columbianus* in the Netherlands, where infections with LPAI were associated with reduced foraging and significant delay in the timing of spring migration [15].

Understanding disease dynamics of LPAI viruses is important also for understanding the emergence and spread of virus strains capable of infecting domestic birds. Specific changes in the hemagglutinin (HA) molecule in H5 and H7 subtypes cause increased virulence in poultry, which can lead to outbreaks of highly pathogenic avian influenza (HPAI) [9], [16], [17]. Once started, such outbreaks can spread regionally in poultry [17], or, as in the case of the ongoing HPAI H5N1 epizootic, revert back into wild birds causing deaths in both domestic and wild bird populations [9], [18]. The capacity for wild waterfowl to disperse HPAI viruses through migration is currently a matter of debate. Experimental data clearly show that dabbling ducks are much less affected by HPAI infection than poultry [19], [20] and one study suggest that ducks previously infected with LPAI can subsequently carry HPAI infections asymptomatically [21]. Data from waterfowl migration (counts, band recoveries, transmitters, loggers etc.) and geographic information have been used to model the capacity of waterfowl to transmit HPAI virus through migration [3], [22]–[25]. Such data could also be used to identify areas of higher risk for HPAI and point out relevant sites for influenza A surveillance. However, crucial knowledge is lacking, for example how virus transmission is related to natal or breeding origin of the reservoir hosts. Patterns of LPAI dynamics indeed vary in time and space; prevalence in waterfowl shows distinctive autumn peaks at major stopover sites in Europe [11], and there is also a large-scale latitudinal gradient of generally higher prevalence rates at northern than at southern sites [10]. Local-scale dynamics of LPAI subtypes may also be involved, demonstrated by variations within- as well as between-year at specific sites [11], [26], [27]. One hypothesis to explain these patterns is that hosts from geographically different breeding areas carry different sets of LPAI subtypes and arrive at staging sites at different times in autumn. To date, a general limitation in bridging the ecology and epidemiology of disease in natural systems has been determining the origin of autumn-staging birds, of which most are immuno-naïve hatch-years on their first migration and therefore cannot be readily banded or sampled in their natal areas.

Banding of popular game species, such as dabbling ducks, results in relatively high recovery rates since a large share ends up in hunters' bags [28]. However, since most banding and hunting of migratory species takes place far from the breeding grounds, recoveries give scant information about natal origin, cf. [29]. These and other biases can be overcome by using intrinsic markers such as stable isotope measurements of body tissues such as feathers [30]. Knowledge about origin based on isotopes could then be used to relate, for instance, the prevalence of certain diseases. In this way, LPAI infection in Bewick's swans as well as avian malaria blood parasites in great reed warblers *Acrocephalus arundinaceus* were found to differ among wintering areas or habitat [31], [32]. One of the more commonly used isotope elements is hydrogen, based on the fact that the relative abundance of deuterium to protium (^2^H/^1^H, measured as δ^2^H) in annual or growing season precipitation is related, at continental scales, largely to latitude and altitude [33]. The correlation between deuterium in feathers (δ^2^H_f_) grown at specific sites and in long-term amount-weighted precipitation at the same sites (δ^2^H_p_) is often strong [34], [35], allowing inferences about where the feather was grown. Feathers are metabolically inert following growth and so the δ^2^H_f_ value conveys information about origin. The recent development of Bayesian assignment approaches to isotope data [36] allows a probabilistic approach to assigning birds to isoscapes including the use of informed priors such as migration directions provided by band recoveries [37].

The aims of the present study were 1) to delineate natal origins of autumn-staging mallards in southern Sweden and 2) to evaluate whether natal origin is related to the variation of influenza A subtypes shed by mallards. We found that birds from different origins had temporal structure in their prevalence of influenza A subtypes. This may have consequences for the likelihood of pathogen transmission throughout the flyway.

## Results

### Recaptured mallards and probabilistic determination of natal grounds

**Figure 1:**
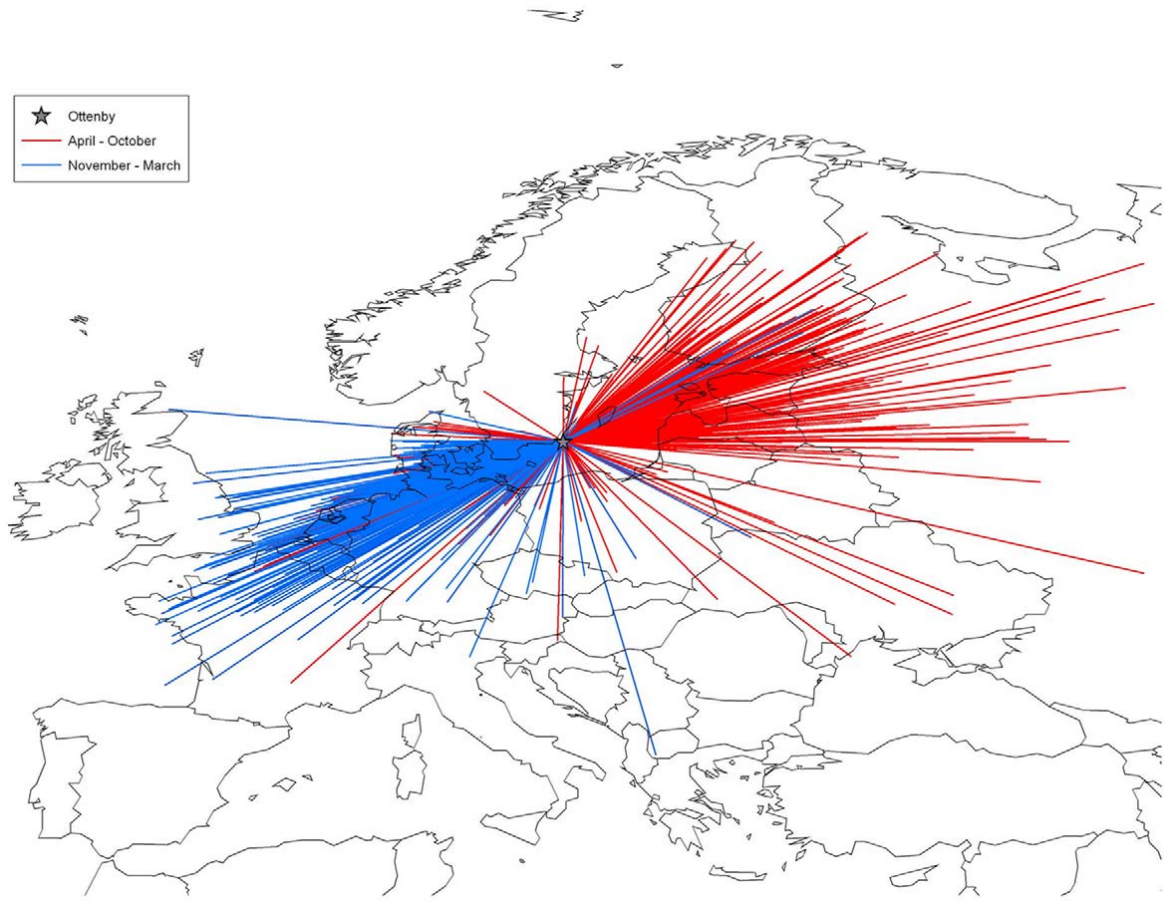
Vectors linking recovery locations of mallards marked at Ottenby 1962–1984 and 2002–2009. Data are divided into different periods representing wintering (November-March; blue) and breeding/migrating (April-October; red) birds.

The mean direction towards the putative breeding grounds for a subset of 49 birds (of the total sample of 924) banded at Ottenby and subsequently re-encountered between April 15 and July 15, was 56° (95% CI 51.7–60.5°; kappa = 13.09). To enlarge the sample, the directions from all mallards re-encountered in any time of the year (Figure 1), were rescaled to fall between 315–135°. Rescaling was accomplished by adding 180° to values falling between 135–315°, if the result was >360°, the remainder after subtracting 360 (i.e. modulo operation [37]). The mean direction for the rescaled data set was very similar to the subset of 49 birds recaptured on breeding grounds (mean direction = 55°, 95% CI 53.8–56.5°; kappa = 7.51). Since the mean direction did not differ between the restricted and the larger data sets (circular Anova F_2,921_ = 0.3, p = 0.7), all 924 directions were used in subsequent calculations since the smaller kappa (an inverse measure of dispersion) is more conservative (implies a wider range of potential directions).

**Figure 2:**
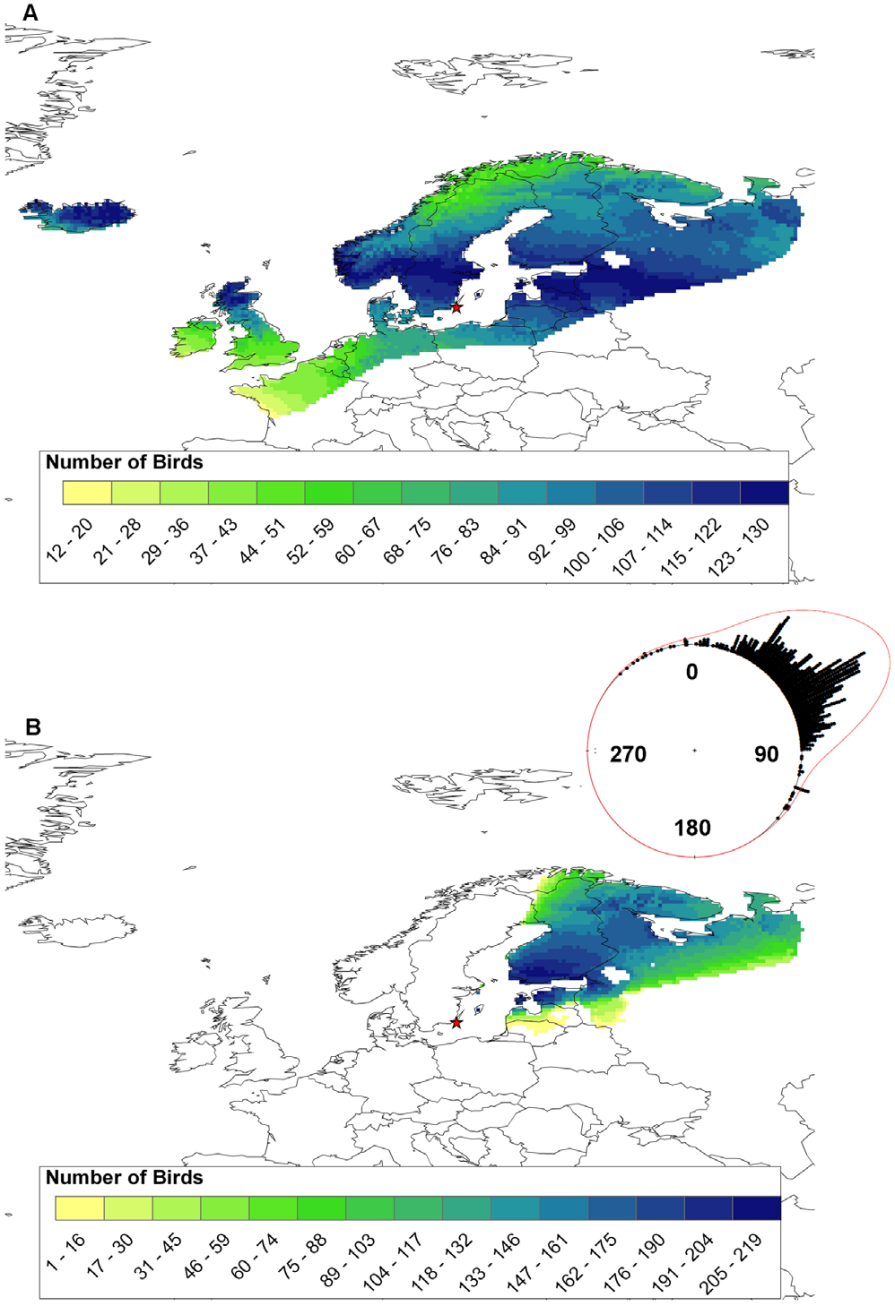
Geographic distribution of assigned natal origins of mallards. Natal origin of 252 hatch-year mallards, sampled at Ottenby (red star) during the autumns in 2004 and 2005, were inferred using A) stable isotope (δ^2^H_f_) analysis of feathers, and B) using combined analysis of δ^2^H_f_ and band recovery data from a larger set of 924 hatch-year mallards. Natal origins were assigned to a GIS based model [33] of stable isotopes in precipitation (δ^2^H_p_), calibrated to reflect expected δ^2^H_f_ via regression (see Methods). Assignments were made using a Bayesian framework incorporating A) spatially explicit likelihood functions based upon δ^2^H_f_, and B) using the same framework but assessing the joint likelihood from A) and spatially explicit prior probabilities estimated by fitting a von Mises distribution to band recovery data (inset on panel B; mean direction = 55°, kappa = 7.51).

Geographic assignments made solely on the basis of δ^2^H_f_ suggest that the majority of mallards staging at Ottenby were isotopically consistent with northern portions of its European range, extending from Iceland, northern UK, through Scandinavia and into Russia (Figure 2A). However, based on a very large set of band recoveries from Ottenby and other sites in southern Scandinavia [29], [38] it is extremely unlikely that mallards staging at Ottenby during autumn migration originate from the northwest, let alone from the west. Prior probabilities based on applying a von Mises distribution to band recovery data (using 2∶1 odds, see Materials and Methods) support this contention (Figure 2B, inset), eliminating many isotopically similar areas as unlikely origins for mallards staging at Ottenby (Figure 2B). Application of prior probabilities suggests that the majority of mallards staging at Ottenby originated from Estonia, southern and central Finland and northwestern Russia near Murmansk and the White Sea (Figure 2B). Similar results were obtained using more conservative odds of 9∶1 in selecting the most likely regions of origin for our samples (Figure S1).

### Temporal trends of origin and influenza A virus infections

#### Feather deuterium

**Figure 3:**
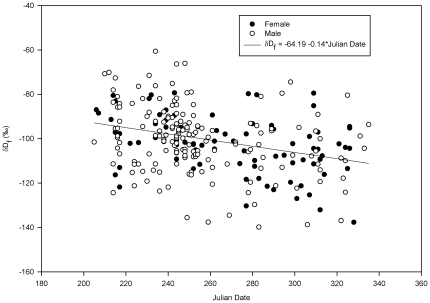
Temporal pattern of δ^2^H_f_ in mallards staging at Ottenby. Data, separated by sex (male and females) are from autumns 2004 and 2005.

The δ^2^H_f_ data ranged from −140 to −61‰. No interactions (2-way and 3-way) in a GLM analysis with δ^2^H_f_ as dependent variable, and sex, year, and date as independent variables were significant (p>0.05). Neither sex (F_1,248_ = 0.43, p = 0.837) nor year were significant factors (F_1,249_ = 2.433, p = 0.120). Date (within-season), however, clearly explained the variation in δ^2^H_f_ (F_1,250_ = 29.568, p<0.001; Figure 3). Parameter estimates in the final model were thus β = −0.14 (SEM = 0.03) for date and β = −64.19 (SEM = 1.69) for the intercept. The parameter estimate for date suggests a chain migration strategy, with mallards staging at Ottenby in the end of the autumn tending to come from farther northeast than birds staging there in early autumn. The parameter estimates indicate that δ^2^H_f_ of mallards staging at Ottenby in late autumn is on average depleted by 19‰ relative to mallards staging there in the early autumn (Figure 3).

**Figure 4:**
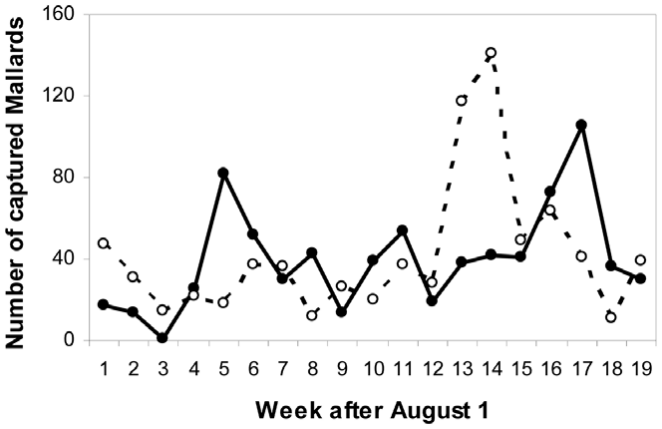
The number of mallards captured at Ottenby in two years. Data are from 2004 (filled symbols and solid line; N = 762) and 2005 (open symbols and dashed line; N = 795), and range from August 1 (starting “week 1”) to December 11 (ending “week 19”).

The number of staging ducks in the Ottenby area correlated with the number of trapped ducks there (J. Waldenström, unpublished data). Peaks in capture rate therefore correspond to influx peaks of ducks, mainly hatch-year birds. In 2004, there were two distinct peaks, one in early (week 5), and one in late autumn (week 17), whereas there was only one clear peak in 2005 (weeks 13–14) (Figure 4). Based on these figures, two autumn periods were defined to be contrasted in terms of δ^2^H_f_ and influenza A virus infection: week 1–8 (hereafter “early period/autumn”) and week 12–19 (hereafter “late period/autumn”).

Comparing δ^2^H_f_ between early and late autumn (N_early_ = 162, N_late_ = 52) by GLM confirmed a period effect (F_1,211_ = 13.361, p<0.001) with feathers having higher δ^2^H values in early (mean = −98.73‰, SEM = 1.04) than in late autumn (mean = −107.68‰, SEM = 2.04). There was a year effect (N_2004_ = 107, N_2005_ = 107, F_1,211_ = 4.742, p = 0.031) with somewhat lower δ^2^H_f_ in 2004 (mean = −103.63‰, SEM = 1.24) than in 2005 (mean = −98.19‰, SEM = 1.43). There were neither significant 2- or 3-way interactions (p>0.05), nor any effect of sex (N_females_ = 150, N_males_ = 64, F_1,210_ = 0.041, p = 0.839).

#### Low-pathogenic influenza A virus

A large proportion of the autumn-staging mallards at Ottenby in 2004 and 2005 was infected with LPAI virus. Among all hatch-year birds captured (August 1 to December 11 in both years) 19.1% (143 of 750) were positive for influenza A, i.e. 17.1% (86 of 503) in 2004 and 23.1% (57 of 247) in 2005. There were small differences in prevalence when autumn periods were contrasted (16.4–19.3%) (i.e. 31 of 189 in early autumn 2004, 41 of 239 in late autumn 2004, and 27 of 140 in early autumn 2005) except for late autumn in 2005, when 36.5% (27 of 74) of the hatch-year birds were infected.

**Figure 5:**
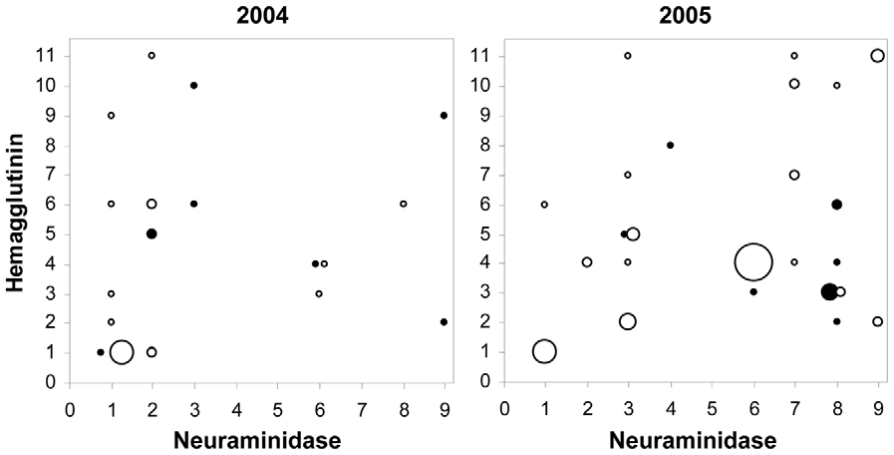
LPAI subtypes in two autumn periods in 2004 and 2005. Early autumn (i.e. period 1: week 1–8 [starting August 1]) are denoted by filled symbols and late autumn (i.e. period 2: week 12–19) by open symbols. Smallest symbols represent one occasion, and bigger symbols with several occasions (relative increase).

In early and late autumn periods, 99 samples out of 270 positive mallards were successfully subtyped (Figure 5). These belonged to either of three age categories, i.e. (1) hatch-year (N = 43), (2) post hatch-year (N = 3), and (3) unaged (N = 53). Altogether, 32 different influenza A virus subtypes were recorded (H1–11; all nine neuroaminidase types except N5, cf. [9]) of which 10 were only found in 2004 and 16 only in 2005. Of the 16 different subtypes detected in 2004, only two (H1N1 and H4N6) occurred in both early and late autumn (seven different subtypes were found in early autumn and 11 in late). The most common (≥10%) subtypes in 2004 were H1N1 (33%) and H1N2 (10%) (Figure 5). In 2005, 22 subtypes were found, again with only two (H3N8 and H5N3) in common for both early and late autumn birds (seven different subtypes were found in early autumn and 17 in late). Once more, H1N1 was among the most common influenza A virus subtypes (13%), together with H4N6 (20%) and H3N8 (10%) (Figure 5). The Sørensen similarity index was 0.22 in 2004 and 0.17 in 2005 contrasting early and late autumn periods, confirming low similarity in subtype occurrence. Similarity was in fact higher between years (0.32) than within (cf. Figure 5).

## Discussion

The temporal structure in LPAI subtype diversity could have several explanations. Since we found a corresponding temporal effect in the natal origin of the mallards, we propose that migrants from less distant natal areas in the early autumn were infected by virus subtypes different from those found in mallards from more distant origins migrating through southern Sweden in late autumn. Data on influenza A virus infections are present from the staging site at Ottenby only, and not from the breeding grounds, which would be necessary to confirm our proposition. Although other factors (e.g. arrival sequence for other influenza A virus hosts, environmental factors etc., as discussed below) may have induced the temporal pattern in subtype diversity, we consider the ‘natal origin hypothesis’ to be a strong candidate. We therefore believe that our results, confirming the previous suggestion that late-arriving and late-staging autumn mallards in the Northwest European flyway come from more distant breeding areas [11], potentially has several important implications for relationships between avian migration and the epidemiology of common pathogens such as the influenza A virus.

The prevalence of influenza A virus in mallards sampled at Ottenby in 2004 and 2005 was similar to those previously reported within the same flyway (i.e. 10–30% [10], [11]). The exception was late autumn in 2005 with particularly high rates (36.5%). This is not extraordinary though, since it is known that prevalence can be highly variable between years and seasons [11]. Long-term surveillance over nearly ten years has shown that the prevalence of this virus in the mallard populations sampled at Ottenby shows a consistent annual pattern, with little or no virus circulation during spring and summer, with peak viral circulation during staging/migration (September to November), and declining prevalence in December [10], [11]. Precise timing of peaks in autumn is, however, variable between years [11]. This is likely due to between-year weather-related differences in the migration time-table; LPAI dynamics is indeed a large-scale phenomenon with migration influencing disease transmission [3], [4].

We found 32 different influenza A virus subtype combinations. Of these, H4N6 viruses seems to occur frequently and are globally widespread in ducks [10]–[12], [39], whereas HIN1 and H3N8 have been reported as common subtypes in Europe [39] and North America [12], respectively. Low similarity (Sørensen's index) between viral subtype diversity in mallards arriving at Ottenby in early *versus* late autumn clearly illustrated within-season shifts in virus subtypes. These within-season differences were in fact higher than those between years, corroborating the pattern in Wallensten et al. [11] who included mallard data from Ottenby in 2002–2004 (not autumn 2004; i.e. a different sample compared to the present study). Twenty of the 39 detected subtypes described by Munster et al. [10] were also found in our study (Sørensen similarity index 0.56 when the two studies are compared). Almost all subtypes that were unique for each study were found only once (i.e. each representing only ca 1% of all subtyped viruses). We acknowledge the potential shortcoming of using also adult and unaged birds, in addition to juveniles, in the analyses of subtype data, in contrast to the analyses on natal origins which included juveniles only. We feel however, that this would be unlikely to induce any bias in our comparisons. In fact, for the juveniles only, there would have been only one subtype in 2004 (H1N1) occurring in both early and late autumn periods, in contrast to two subtypes in 2004 and two in 2005 in common for the periods with data on adult and unaged birds. Interpretations would thus have been the same, but with lower statistical power due to less data.

The shedding time of the influenza A virus is relatively short in naturally infected mallards, normally lasting 3–8 days in autumn birds at Ottenby [14]. However, from experimental infections it seems that primary infection can last longer, with virus shedding up to two or three weeks [40], [41]. Moreover, mallards are frequently reinfected by different subtypes during their stopover [14]. Virus prevalence and diversity in the sampled staging populations will therefore be a function of virus-related factors, such as the number and prevalence of virus subtypes present, their infectivity and virulence, and host-related factors such as animal numbers, animal condition, pre-existing immunity and stopover duration. Several of these factors could also be influenced by seasonal factors such as temperature, productivity or day length. The large turnover of virus subtypes within seasons suggests that most subtypes are not capable of remaining in circulation in the staging populations of mallards at Ottenby for extended periods of time. Changes in subtypes could then theoretically be explained by cohorts of ducks bringing influenza A virus specific to their area of origin to the sampled staging site. However, although numbers of trapped mallards showed significant variation within seasons (Figure 4), suggesting distinct influx and emigration of birds, there are also large numbers of other susceptible host species in the area, such as swans, geese, other dabbling ducks, diving ducks, gulls and waders. Some species within these groups are captured at Ottenby during autumn, but in lower numbers: for example Eurasian wigeon (*Anas penelope*, 343 samples, 32 positive) common teal (*Anas crecca,* 124 samples, 24 positive) and northern pintails (*Anas acuta*, 58 samples, 3 positives). The limited available data from these species do not suggest marked deviations in the timing of influenza A virus prevalence peaks compared to mallards, but timing of migration may differ; e.g. the peak of common teals is usually one month before that of mallard [42]. The role of non-mallard waterbirds in influenza A virus transmission is still poorly understood, except for gulls, which tend to carry gull-specific virus subtypes H13 and H16 [43]. However, two recent studies on migratory geese in Europe (white-fronted goose *Anser albifrons* and pink-footed goose *Anser brachyrhynchus*) indicate that most transmission is primarily from ducks to geese, rather than the geese being significant reservoirs themselves [17], [44], [45]. At our study site, the mallard is the most common dabbling duck species during nearly the entire autumn period, and also the most sampled species. Increased trapping efforts for other species are warranted to determine the potential influence of non-mallard waterbirds to influenza A virus dynamics in this system.

Abiotic factors, such as pH, salinity and temperature, certainly affect virus tenacity in the environment [46]. If these factors vary temporally, or, if there are subtype-specific differences in virus persistence [47], [48], they could affect the rate of subtype turnover in hosts that are utilizing a common environment.. Our study was conducted in a seasonal environment with progressively shorter days and lower water temperatures in the autumn. However, a drop in prevalence rates is normally not seen until ice formation. In laboratory studies, virus tenacity in water range from several weeks to years depending on virus and experimental setup [49]. Field data from North America suggest that virus can persist in lakes over the course of a winter and infect birds in spring [48]. Between-year persistence of virus is not clearly evident in our study system, as naïve lure ducks, introduced to the duck trap at Ottenby in the spring to attract wild ducks, do not tend to be infected until migratory birds have appeared in larger numbers (J. Waldenström unpublished data). At subtype level, the available data suggest that most subtypes occur in rather short time frames, indicative of smaller, passing outbreaks rather than a constant circulation that would be expected if infection from an abiotic water reservoir occurred frequently.

The subtype diversity was higher in late autumn than in early autumn in both years. We speculate that mallards arriving in late autumn not only bring subtypes specific to their natal area, but also pick up “new” ones from conspecifics along the migration route, leading to higher subtype diversity on the population level. An alternative explanation for different patterns of subtype occurrence in early *versus* late autumn would be that immunity against certain subtypes will change the subtype composition over the season. In experimental studies, infected mallards show homosubtypic immunity (short-term [21], [40], as well as long-term [50]), and even heterosubtypic effects, where partial or complete immunity derived from the infection of a certain subtype may be active also against other subtypes [21], [40].

Waterfowl, and especially mallards, are among the most frequently recovered species in European band recovery efforts [29]. Our study shows that when combined with migration directions based on band recoveries, δ^2^H_f_ measurements of mallards emphasize the importance of natal areas in Finland and northwestern Russia. This is consistent with previous studies [11], [29] and suggests further that almost all hatch-year mallards staging at Ottenby in autumn are foreign birds, cf. [11]. The likely linkage between the epidemiology of influenza A virus, mallard migration and natal area as suggested by our data, lead to possibilities in predicting influenza virus dynamics within a flyway. In more general terms, migration has indeed been shown to be important for the dynamics of several common pathogens in the wild (e.g. Lyme disease, West Nile virus, and avian malaria parasites) [5], [31], and identifying spatiotemporal transmission routes of pathogens is therefore of great and wide interest. Naturally, a prerequisite of this approach is the ability to reliably link an individual, or sets of co-occurring individuals, to distinct populations with known natal, stopover and wintering areas. In this respect, the mallard seem to be a good model species given its large population size, a distribution covering large geographical areas with varying baseline isotopic signatures, and its status as a common game species. The δ^2^H measurements presented here co-varied with temporal trends in timing of migration and the virus subtypes infecting them. Clearly, a structured sampling of feathers from mallards at breeding sites across the distribution is warranted, providing a refined feather isoscape to which samples can be probabilistically assigned. Recent outbreaks of HPAI H5N1 in Asia, Europe and Africa, provide a concrete example of how such data could prove useful. Such outbreaks are potentially devastating to the poultry industry, and pose risks to human health. Thus, improved knowledge of migratory connectivity between populations and timing of migration in relation to climatic factors would allow improved prediction of disease transmission and potential for HPAI outbreaks, facilitating risk assessment and management in Europe and elsewhere.

## Materials and Methods

### Ethics Statement

All handling of birds was done in accordance with Swedish legislation of animal welfare, and ethical approval was obtained from the Linköping Animal Research Ethics Board (permit number 43-09 and 83-10).

### Trapping and sampling

The study was performed at a major stopover site (Ottenby in southeast Sweden, 56°13′N 16°27′E; http://www.ofstn.ottenby.se) centrally located in the Northwest European flyway [51], [52], ranging from the Kola Peninsula to southwestern France. Mallards have been captured at Ottenby since 1962 in a stationary baited (grain) duck trap bordering the Baltic Sea. Trapping was discontinued entirely from 1985 to 2001 and briefly in some other years for repairs. Otherwise it has been in operation daily from March to December (or until ice forms). Mallards were collected daily for banding and measurements, after which they were immediately released. Data used in the present study include age, sex, deuterium fractions analyzed from feathers (described below), as well as prevalence and subtypes of the influenza A virus. Influenza A sampling was done by swabbing the cloaca or fresh faeces. Infection of influenza A virus was detected by a real-time PCR assay targeted at the influenza A virus matrix gene [53]. Positive samples were later propagated in embryonated chicken eggs to isolate virus and to determine virus subtypes. For a more detailed description of duck trapping and laboratory analyses, see Latorre-Margalef et al. [14].

Data considered in the analyses described below (except band recovery data) are from two autumns (2004 and 2005) and concern mallards staging on their way to wintering areas farther southwest in the flyway. A fraction of the birds present in late autumn may stay in the Ottenby area to winter if weather permits. The breeding population of mallards in the Ottenby area is very small; the vast majority of sampled staging ducks originate from areas farther north and northeast in the flyway, cf. [29].

Hatch-year mallards only were considered in most analyses. However, post hatch-year as well as unaged birds were considered in a few analyses (see below and in result section) to enlarge the samples.

### Natal origin analyses based on stable isotopes and band recoveries

#### Deuterium fractions in feathers

Vane from the (distal) tip of one tail feather from 252 hatch-year mallards (170 males [89 in 2004 and 81 in 2005] and 82 females [42 in 2004 and 40 in 2005]) was collected from the end of July to early December. In order to include only feathers grown in natal areas, only unmolted feathers were used. Hatch-year birds molt their tail feathers during the autumn starting as early as September [42]. Deuterium fractions were analysed at the Environment Canada stable isotope facility in Saskatoon, Canada. External oils and dirt were removed from the surfaces of feathers using multiple 2∶1 (v/v) chloroform: methanol rinses followed by drying in a fume-hood overnight. Stable isotope ratios of cleaned feather material, excluding the rachis, were determined using an elemental analyzer coupled to a Continuous-Flow Isotope Ratio Mass Spectrometer (CF-IRMS). In order to reduce error associated with the exchange of hydrogen between feather material and ambient air for δ^2^H assays, samples and isotopic standards were treated using the comparative equilibration method described by Wassenaar and Hobson [54]. Hydrogen isotope samples (350 µg) were pyrolyzed and analyzed along with three calibrated, in-house keratin laboratory standards; Cow Hoof Standard (CHS) (δ^2^H = −187‰), Bowhead Whale Baleen (BWB) (δ^2^H = −108‰) and Chicken Feather Standard (CFS) (δ^2^H = −147.4‰), all with SD values better than ±1.0‰ for within-run replicate measurements. Measurements are reported in standard δ-notation in parts per thousand (‰) deviation from the Vienna Standard Mean Ocean Water (VSMOW).

#### Recovery data and probabilistic determination of natal grounds

Recapture data (both live and dead) from mallards banded at Ottenby 1962–1984 and 2002–2009 were used to assess migratory direction, which was calculated using the great-circle bearing between all banding and recapture locations. Migration direction was analyzed using circular statistics based on the von Mises distribution [55]. Only hatch-year birds marked July–December were considered. Ducks were excluded if they qualified for warning codes according to the EURING protocol (e.g. abnormal plumage, morphology, or injury [56]), and if they were recaptured within 100 km from Ottenby. In total, 924 (317 females and 607 males) mallards were included in the probabilistic analysis of natal grounds.

Mallard origins were depicted probabilistically using a likelihood-based assignment approach. This involved the creation of a feather δ^2^H isoscape using a precipitation-to-feather calibration algorithm (δD_f_ = −31.6+0.93 δD_p_) based on data from known-origin of Lesser Scaup *Aythya affinis* feathers [57], [58]. The δ^2^H_p_ isoscape was created by converting a GIS-based model of expected amount-weighted growing-season δ^2^H_p_ (hereafter δD_p_ [33]). We estimated the likelihood that a cell within the δ^2^H_f_ isoscape represented a potential origin for a sample by using a normal probability density function to estimate the likelihood function based upon the observed δ^2^H_f_ [37], [59]. Next, we assessed the posterior likelihood by applying a von Mises probability density function fit to the aforementioned band recovery data, and incorporated these as spatially explicit prior probabilities using Baye's Theorem to assess the joint likelihoods [37].

The likely origins of sampled mallards were depicted by assigning individuals to the δ^2^H_f_ isoscape one at a time. Following Hobson et al. [59], this was accomplished by first determining the odds that an assigned origin was correct relative to the odds it was incorrect. Based on 2∶1 odds that a given bird had truly originated from within the range defined by the odds ratio, we recoded the set of raster cells that defined the upper 67% of estimated ‘probabilities of origin’ and coded those as 1, and all others as 0, resulting in one binary map per assigned individual. The results of the individual assignments were summed over all individuals by addition of the surfaces (details available in Hobson et al. [59] and Van Wilgenburg and Hobson [37]). For comparative purposes, we also present assignments made using 9∶1 odds, created by selecting the upper 90% of the estimated ‘probabilities of origin’. Geographic assignments to origin were done using functions within the R statistical computing environment (R Development Core Team 2009) using the ‘raster’ package.

### Temporal trends of origin and influenza A virus infections

Earlier observations indicate that there are more or less distinct influx peaks of mallards at Ottenby in autumn (trapped as well as staging birds; J. Waldenström unpublished data). Hypothesizing that hatch-year mallards in each of these peaks originated from different areas, data from ducks belonging to these peaks were compared. Accordingly, a GLM analysis, performed in SPSS 18.0, was used to study temporal patterns in δ^2^H_f_. Differences in the diversity of influenza A virus subtypes, for mallards in any of the three age categories, were evaluated descriptively, as well as with using the Sørensen similarity index (range 0–1 [60]).

## Supporting Information

Figure S1 **Geographic distribution of assigned natal origins of mallards using 9∶1 odds to classify likely versus unlikely origins for each sample.**
(TIF)

## References

[1] Alerstam T (1990). Bird migration.

[2] Newton I (2010). Bird Migration.

[3] Kilpatrick AM, Chmura AA, Gibbons DW, Fleischer RC, Marra PP (2006). Predicting the global spread of H5N1 avian influenza.. Proc Natl Acad Sci U S A.

[4] Marra PP, Griffing S, Caffrey C, Kilpatrick AM, McLean R (2004). West Nile virus and wildlife.. Bioscience.

[5] Altizer S, Bartel R, Han BA (2011). Animal migration and infectious disease risk.. Science.

[6] Webster MS, Marra PP, Haig SM, Bensch S, Holmes RT (2002). Links between worlds: unraveling migratory connectivity.. Trends Ecol Evol.

[7] Procházka P, Stokke BG, Jensen H, Fainová D, Bellinvia E (2011). Low genetic differentiation among reed warbler *Acrocephalus scirpaceus* populations across Europe.. J Avian Biol.

[8] Slatkin M (1985). Gene flow in natural populations.. Annu Rev Ecol Syst.

[9] Olsen B, Munster VJ, Wallensten A, Waldenström J, Osterhaus ADME (2006). Global patterns of influenza A virus in wild birds.. Science.

[10] Munster VJ, Baas C, Lexmond P, Waldenström J, Wallensten A (2007). Spatial, temporal, and species variation in prevalence of influenza A viruses in wild migratory birds.. PLoS Pathog.

[11] Wallensten A, Munster VJ, Latorre-Margalef N, Brytting M, Elmberg J (2007). Surveillance of influenza A virus in migratory waterfowl in northern Europe.. Emerg Infect Dis.

[12] Krauss S, Walker D, Pryor SP, Niles L, Chenghong L (2004). Influenza A viruses of migrating wild aquatic birds in North America.. Vector Borne Zoonotic Dis.

[13] Daoust PY, Kibenge FSB, Fouchier RAM, van de Bildt MWG, van Riel D (2011). Replication of low pathogenic avian influenza virus in naturally infected Mallard ducks (*Anas Platyrhynchos*) causes no morphologic lesions.. J Wildl Dis.

[14] Latorre-Margalef N, Gunnarsson G, Munster VJ, Fouchier RAM, Osterhaus ADME (2009). Effects of influenza A virus infection on migrating mallard ducks.. Proc R Soc Lond B Biol Sci.

[15] van Gils JA, Munster VJ, Radersma R, Liefhebber D, Fouchier RAM (2007). Hampered foraging and migratory performance in swans infected with low-pathogenic avian influenza A virus.. PLoS ONE.

[16] Capua I, Alexander DJ (2007). Avian influenza in birds - a moving target.. Influenza.

[17] Capua I, Mutinelli F, Pozza MD, Donatelli I, Puzelli S (2002). The 1999–2000 avian influenza (H7N1) epidemic in Italy: veterinary and human health implications.. Acta Trop.

[18] Chen H, Smith GJ, Zhang SY, Qin K, Wang J (2005). Avian flu: H5N1 virus outbreak in migratory waterfowl.. Nature.

[19] Keawcharoen J, van Riel D, van Amerongen G, Bestebroer T, Beyer WE (2008). Wild ducks as long-distance vectors of highly pathogenic avian influenza virus (H5N1).. Emerg Infect Dis.

[20] Sturm-Ramirez KM, Hulse-Post DJ, Govorkova EA, Humberd J, Seiler P (2005). Are ducks contributing to the endemicity of highly pathogenic H5N1 influenza virus in Asia?. J Virol.

[21] Fereidouni SR, Starick E, Beer M, Wilking H, Kalthoff D (2009). Highly pathogenic avian influenza virus infection of mallards with homo- and heterosubtypic immunity induced by low pathogenic avian influenza viruses.. PLoS ONE.

[22] Brochet AL, Guillemain M, Lebarbenchon C, Simon G, Fritz H (2009). The potential distance of highly pathogenic avian influenza virus dispersal by mallard, common teal and eurasian pochard.. EcoHealth.

[23] Lebarbenchon C, Albespy F, Brochet A-L, Grandhomme V, Renaud F (2009). Spread of avian influenza viruses by Common Teal (*Anas crecca*) in Europe.. PLoS ONE.

[24] Prosser DJ, Takekawa JY, Newman SH, Yan B, Douglas DC (2009). Satellite-marked waterfowl reveal migratory connection between H5N1 outbreak areas in China and Mongolia.. Ibis.

[25] Reperant LA, Fučkar NS, Osterhaus ADME, Dobson AP, Kuiken T (2010). Spatial and temporal association of outbreaks of H5N1 influenza virus infection in wild birds with the 0°C isotherm.. PLoS Pathog.

[26] Globig A, Baumer A, Revilla-Fernández S, Beer M, Wodak E (2009). Ducks as sentinels for avian influenza in wild birds.. Emerg Infect Dis.

[27] Jahangir A, Watanabe Y, Chinen O, Yamazaki S, Sakai K (2008). Surveillance of avian influenza viruses in Northern Pintails (*Anas acuta*) in Tohoku district, Japan.. Avian Dis.

[28] Hirschfeld A, Heyd A (2005). Mortality of migratory birds caused by hunting in Europe: bag statistics and proposals for the conservation of birds and animal welfare.. Berichte zum Vogelschutz.

[29] Fransson T, Pettersson J (2001). Svensk ringmärkningsatlas [Swedish bird ringing atlas].

[30] Hobson KA, Wassenaar LI (2008). Tracking animal migration using stable isotopes.

[31] Yohannes E, Hansson B, Lee RW, Waldenström J, Westerdahl H (2008). Isotope signatures in winter moulted feathers predict malaria prevalence in a breeding avian host.. Oecologia.

[32] Hoye BJ, Fouchier RAM, Klaassen M (2012). Host behaviour and physiology underpin individual variation in avian influenza virus infection in migratory Bewick's swans.. Proc R Soc Lond B Biol Sci.

[33] Bowen GJ, Wassenaar LI, Hobson KA (2005). Global application of stable hydrogen and oxygen isotopes to wildlife forensics.. Oecologia.

[34] Chamberlain CP, Blum JD, Holmes RT, Feng X, Sherry TW (1997). The use of isotope tracers for identifying populations of migratory birds.. Oecologia.

[35] Hobson KA, Wassenaar LI (1997). Linking breeding and wintering grounds of neotropical migrant songbirds using stable hydrogen isotopic analysis of feathers.. Oecologia.

[36] Wunder MB, West JB, Bowen GJ, Dawson TE, Tu KP (2010). Using isoscapes to model probability surfaces for determining geographic origins.. Isoscapes: understanding movement, pattern, and process on earth through isotope mapping.

[37] Van Wilgenburg SL, Hobson KA (2011). Combining stable-isotope (δD) and band recovery data to improve probabilistic assignment of migratory birds to origin.. Ecol Appl.

[38] Bakken V, Runde O, Tjørve E (2003). Norwegian bird ringing atlas.

[39] Süss J, Schäfer J, Sinnecker H, Webster RG (1994). Influenza virus subtypes in aquatic birds of eastern Germany.. Arch Virol.

[40] Jourdain E, Gunnarsson G, Wahlgren J, Latorre-Margalef N, Bröjer C (2010). Influenza virus in a natural host, the mallard: experimental infection data.. PLoS ONE.

[41] Costa TP, Brown JD, Howerth EW, Stallknecht DE (2010). The effect of age on avian influenza viral shedding in mallards (*Anas platyrhynchos*).. Avian Dis.

[42] Cramp S, Simmons KEL, Cramp S, Simmons KEL (1977). The birds of the western Palearctic.

[43] Fouchier RAM, Munster V, Wallensten A, Bestebroer TM, Herfst S (2005). Characterization of a novel Iinfluenza A virus hemagglutinin subtype (H16) obtained from black-headed gulls.. J Virol.

[44] Kleijn D, Munster VJ, Ebbinge BS, Jonkers DA, Müskens GJDM (2010). Dynamics and ecological consequences of avian influenza virus infection in greater white-fronted geese in their winter staging areas.. Proc R Soc Lond B Biol Sci.

[45] Hoye BJ, Munster VJ, Nishiura H, Fouchier RAM, Madsen J (2011). Reconstructing an annual cycle of interaction: natural infection and antibody dynamics to avian influenza along a migratory flyway.. Oikos.

[46] Brown JD, Goekjian G, Poulson R, Valeika S, Stallknecht DE (2009). Avian influenza virus in water: Infectivity is dependent on pH, salinity and temperature.. Vet Microbiol.

[47] Brown JD, Swayne DE, Cooper RJ, Burns RE, Stallknecht DE (2007). Persistence of H5 and H7 avian influenza viruses in water.. Avian Dis.

[48] Lebarbenchon C, Yang M, Keeler SP, Ramakrishnan MA, Brown JD (2011). Viral replication, persistence in water and genetic characterization of two influenza A viruses isolated from surface water.. PLoS ONE.

[49] Stallknecht DE, Goekjian VH, Wilcox BR, Poulson RL, Brown JD (2010). Avian influenza virus in aquatic habitats: what do we need to learn?. Avian Dis.

[50] Fereidouni SR, Grund C, Häuslaigner R, Lange E, Wilking H (2010). Dynamics of specific antibody responses induced in mallards after infection by or immunization with low pathogenicity avian influenza viruses.. Avian Dis.

[51] Guillemain M, Sadoul N, Simon G (2005). European flyway permeability and abmigration in Teal *Anas crecca*, an analysis based on ringing recoveries.. Ibis.

[52] Scott DA, Rose PM (1996). Atlas of Anatidae populations in Africa and Western Eurasia.

[53] Spackman E, Senne DA, Myers TJ, Bulaga LL, Garber LP (2002). Development of a real-time reverse transcriptase PCR assay for type A influenza virus and the avian H5 and H7 hemagglutinin subtypes.. J Clin Microbiol.

[54] Wassenaar LI, Hobson KA (2006). Stable hydrogen isotope heterogeneity in biological tissues: isotope-ratio mass spectrometry and migratory wildlife sampling strategies.. Rapid Commun Mass Spectrom.

[55] Fisher NI (1995). Statistical analysis of circular data.

[56] Speek G, Clark JA, Rohde Z, Wassenaar RD, Van Noordwijk AJ (2001). The EURING exchange-code 2000.

[57] Clark RG, Hobson KA, Wassenaar LI (2009). Corrigendum - Geographic variation in the isotopic (δD, δ13C, δ15N, δ34S) composition of feathers and claws from lesser scaup and northern pintail: implications for studies of migratory connectivity.. Can J Zool.

[58] Clark RG, Hobson KA, Wassenaar LI (2006). Geographic variation in the isotopic (δD, δ13C, δ15N, δ34S) composition of feathers and claws from lesser scaup and northern pintail: implications for studies of migratory connectivity.. Can J Zool.

[59] Hobson KA, Wunder MB, Van Wilgenburg SL, Clark RG, Wassenaar LI (2009). A method for investigating population declines of migratory birds using stable isotopes: origins of harvested lesser scaup in North America.. PLoS ONE.

[60] Sørensen T (1948). A method of establishing groups of equal amplitude in plant sociology based on similarity of species and its application to analyses of the vegetation on Danish commons.. Biologiske Skrifter.

