# Supplementary figures and images for: Disease Dynamics and Bird Migration—Linking Mallards Anas platyrhynchos and Subtype Diversity of the Influenza A Virus in Time and Space
Source: PLoS One. 2012 Apr 20;7(4):e35679. doi: 10.1371/journal.pone.0035679 (PMC3335010; doi:10.1371/journal.pone.0035679)

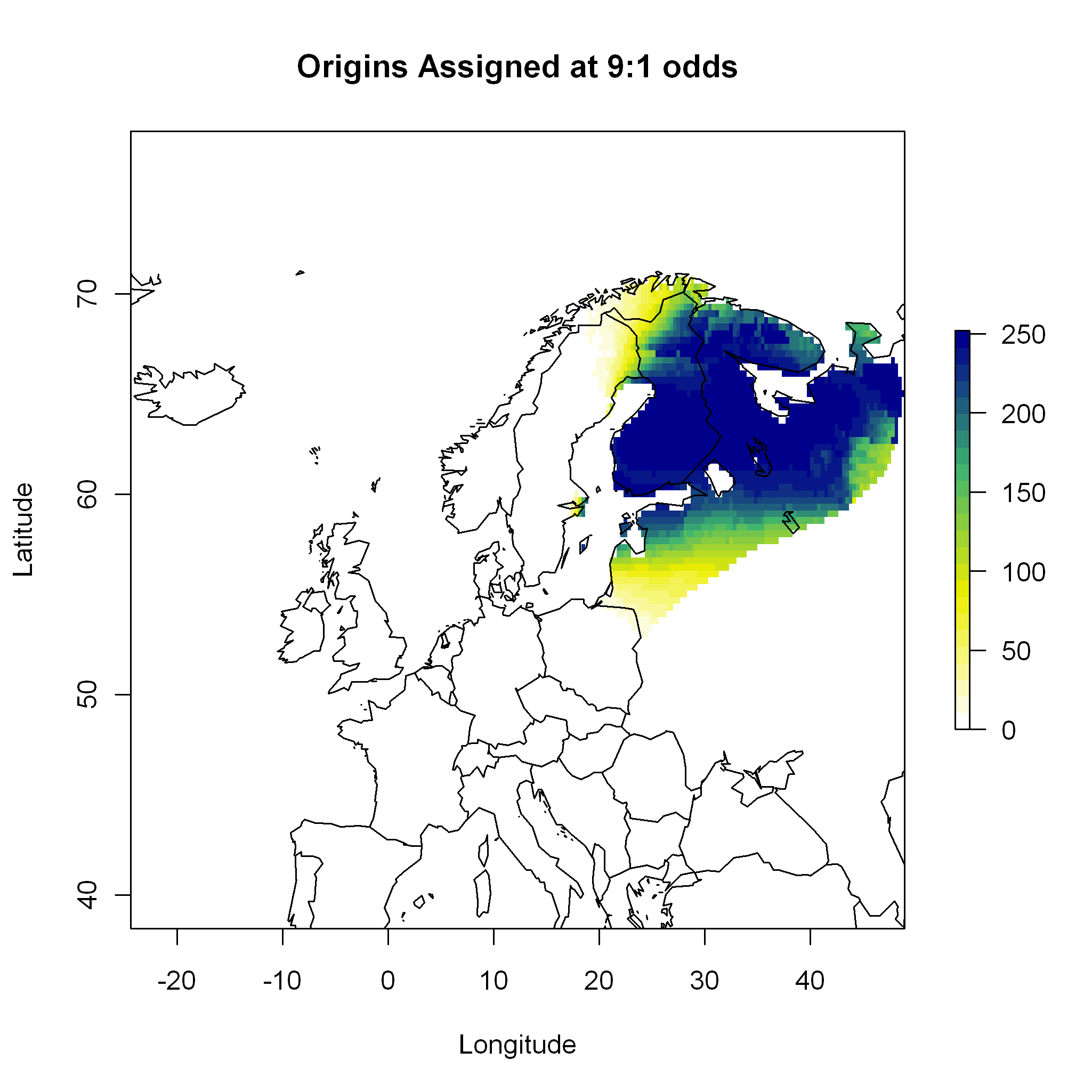

Supplement: Figure S1 — Geographic distribution of assigned natal origins of mallards using 9∶1 odds to classify likely versus unlikely origins for each sample. (TIF) [file pone.0035679.s001.tif]
